# Editorial: Hormonal crosstalk on the regulation of stress responses

**DOI:** 10.3389/fpls.2022.1022143

**Published:** 2022-09-13

**Authors:** Tae-Hwan Kim, Bok-Rye Lee, Md Tabibul Islam, Jean-Christophe Avice

**Affiliations:** ^1^Grassland Science Laboratory, Department of Animal Science, Institute of Agricultural Science and Technology, College of Agriculture and Life Science, Chonnam National University, Gwangju, South Korea; ^2^Institute of Environmentally-Friendly Agriculture (IEFA), Chonnam National University, Gwangju, South Korea; ^3^Alson H. Smith Jr. Agricultural Research and Extension Center, School of Plant and Environmental Sciences, Virginia Tech, Winchester, VA, United States; ^4^Normandie Univ, UNICAEN, INRAE, UMR EVA, SFR Normandie Végétal FED4277, Esplanade de la Paix, Caen, France

**Keywords:** phytohormones, abiotic and biotic stress, stress tolerance mechanism, regulatory mechanisms, hormonal crosstalk, signaling pathway

Plant biotic and abiotic stress poses significant challenges to the agroecosystem in the age of rapid global climate change, thus affecting crop growth and posing a serious risk to agricultural productivity and food security worldwide. Several studies have demonstrated that plant stresses trigger the production of phytohormones leading to an alteration in their balance, which contributes to plant adaption to numerous external stimuli. Although significant progress has been made in the characterization of stress-triggered changes in hormone metabolism in plants, many unknowns remain. This Research Topic reflects the latest updates on hormonal regulation of the complex signaling networks and metabolic pathways and provides future perspectives on the hormonal crosstalk in regulating stress responses and tolerance mechanisms.

Exogenous hormone applications may improve abiotic and biotic stress tolerance by regulating reactive oxygen species (ROS), antioxidant enzyme activity, photosynthesis, soluble sugars, and nitrogen metabolism. Nagar et al. examined the role of a plant hormone gibberellic acid (GA) in terminal heat stress tolerance of wheat genotypes upon spraying GA_3_ or its biosynthesis inhibitor paclobutrazol (PBZ). The authors found that exogenous GA_3_ did not increase thermotolerance, whereas PBZ's application enhanced thermotolerance by increasing antioxidant enzyme activity and photosynthesis, and decreasing lipid peroxidation and ion leakage under heat stress These results suggest that the thermotolerance is closely associated with alternative mechanisms caused by PBZ application but not inhibition of GA biosynthesis. Within the same species, Kaya et al. explored the effects of the combined application of another plant hormone, methyl jasmonate (Me-JA) and nitric oxide (NO)–donor sodium nitroprusside (SNP) in cadmium (Cd) stress mitigation. The results revealed that exogenously applied Me-JA and SNP, either alone or combined, improved nitrogen metabolism in plants grown under Cd stress. Compared to individual applications of Me-JA or SNP, the combined application resulted in more prominent plant growth-promoting effects and Cd level reduction in plant tissues, implying synergistic effects of the two compounds in alleviating Cd toxicity. Additionally, Zhang, Chen, et al. investigated exogenous brassinolide (BR)-induced calcium nitrate stress tolerance in tomato through proteomics analysis, uncovering improved photosynthesis and antioxidant metabolism, and reduced ROS and lipid peroxidation. A study by He et al. implicated the small auxin up-regulated RNA (SAUR) family member *AtSAUR32* in abscisic acid (ABA) signaling under drought stress. *AtSAUR32* participated in drought stress tolerance by regulating transcription factors (DREB, WRKY, and NAC) in ABA-independent signaling pathway and by interacting clade-A PP2Cs proteins in ABA-dependent signaling pathway.

Other research articles in this collection evaluated the impact of exogenous compounds like melatonin, cytokinin 6-benzyladenine (6-BA), and diethyl aminoethyl hexanoate (DA-6) in regulating plant responses to environmental signals. The work by Zhang, Fan, et al. expands our knowledge about the molecular mechanism of melatonin in regulating the cotton response to salt stress using RNA-seq analysis. Exogenous melatonin activated antioxidant enzyme activity and Ca^2+^ signal transduction, which in turn reduced excess production of ROS. In addition, expression of transcription factors (AP2/ERF-ERF, WRKY, NAC, C2H2, etc.) and redox-related genes was upregulated by melatonin under salt stress. The obtained data suggest that melatonin plays a crucial role in regulating the complex network for the salt stress tolerance. In another work published in this research collection, Wang et al. showed the effects of the exogenous application of synthetic 6-BA on plant growth under waterlogging stress in waxy corn. Exogenous 6-BA alleviated the increase of chlorosis, necrosis, ROS and membrane lipid peroxidation by activating antioxidant enzyme activities (ascorbate peroxidase, glutathione reductase, and dehydroascorbate reductase) and the ascorbate-glutathione cycle system in waterlogging-stressed waxy corn, consequently improving waterlogging stress tolerance. Similarly, Hassan et al. showed the pretreatment effect of DA-6 on seed germination of white clover under drought stress. DA-6 pretreatment enhanced indole-3-acetic acid, cytokinin and GA content, but suppressed ABA content, leading to an increase in the ROS scavenging system, osmotic adjustment, dehydrin protein and transcript accumulation.

Furthermore, Aslam et al. reviewed the roles of jasmonate (JA), Ca^2+^ and glutathione (GSH) in abiotic stress tolerance, and their interconnected signaling pathways. Ca^2+^ signaling leads to JA biosynthesis and GSH accumulation, thereby maintaining cellular homeostasis and consequently improving abiotic stress tolerance. This interaction between the JA, Ca^2+^, and GSH represents a novel abiotic stress tolerance mechanism in plants. In the other review article in this collection, Singhal et al. discussed the crosstalk of important signaling compounds (NO, hydrogen sulfide, hydrogen peroxide, Ca^2+^, and ROS) and phytohormones, and their role in salinity stress tolerance. Further, the recent advances are described in integrative multi-omics approaches, which are crucial to leverage to better understand the molecular basis of salinity tolerance and develop new promising salt-tolerant varieties.

And the final article in this collection sheds fresh light on ferroptosis, i.e., iron-dependent-cell death in plants (Dixon et al., 2012). Ferroptosis is mostly regulated by ROS, GSH and Fe^2+^ levels under abiotic and biotic stress (Dixon and Stockwell, 2019). The study by Riyazuddin and Gupta suggests a potential association between gaseous plant hormone ethylene and ferroptosis.

Overall, these articles provide illustrative examples of recent advances in the research area of hormone crosstalk and highlight the complexity of connections between key signaling and metabolic pathways in plants. We hope that these compiled articles will give a new insight into this topic and useful information to expand the research area in the future.

## Author contributions

All authors listed have made a substantial, direct, and intellectual contribution to the work and approved it for publication.

## Conflict of interest

The authors declare that the research was conducted in the absence of any commercial or financial relationships that could be construed as a potential conflict of interest.

## Publisher's note

All claims expressed in this article are solely those of the authors and do not necessarily represent those of their affiliated organizations, or those of the publisher, the editors and the reviewers. Any product that may be evaluated in this article, or claim that may be made by its manufacturer, is not guaranteed or endorsed by the publisher.
